# Occupational acute argon gas poisoning: A case report

**DOI:** 10.1097/MD.0000000000030491

**Published:** 2022-09-09

**Authors:** Yongkai Li, Jianzhong Yang

**Affiliations:** a Emergency Trauma Center, the First Affiliated Hospital of Xinjiang Medical University, Urumqi, China.

**Keywords:** argon gas, asphyxiating gas, poisoning

## Abstract

Intentional or unintentional exposure to asphyxiating gases is a significant public health concern worldwide. Argon poisoning is fatal, and its onset is primarily due to neurological damage.

**Patient concerns:**

A 22-year-old man was admitted to the hospital for argon gas poisoning. While working in a plant containing argon gas, he suddenly lost consciousness, recovered consciousness slightly after on-site treatment, answered questions, and had impaired memory, sensory dullness, normal cognition, and symptoms of dizziness and headache.

**Diagnosis:**

Asphyxiating gas poisoning (argon gas poisoning), metabolic encephalopathy, and hepatic insufficiency.

**Interventions:**

Immediately after admission, the patient was treated with nasal cannula oxygen 3 L/min and hyperbaric oxygen therapy once a day. Mecobalamin tablets 500 μg were given orally 3 times a day. Oral Ginkgo biloba extract tablets 40 mg 3 times a day.

**Outcome:**

The patient was discharged after treatment with hyperbaric oxygen therapy and nerve-nourishing drugs, with no discomfort, clear consciousness, and good memory, and was followed up by telephone for 2 consecutive months, and the patient is now in good condition with no discomfort.

**Lesson:**

This case describes the pathogenesis, neurological damage, and rescue process of argon gas poisoning. Argon poisoning was found to damage bilateral cerebellar hemispheres and bilateral hippocampal regions, affecting the patient’s consciousness and memory, and was found to cause abnormal liver function and heart rate disorders.

## 1. Introduction

Asphyxiating gas is a non-toxic or low-toxic gas that reduces the normal concentration of oxygen in the air. People in high concentrations of asphyxiating gases or breathing a large amount of asphyxiating gases, which makes the body inhale insufficient oxygen concentration, can lead to death by asphyxiation. Since most asphyxiating gases are inert and odorless, high concentrations of asphyxiating gases are difficult to notice. Common asphyxiating gases include methane, nitrogen, argon, helium, butane, and propane. These gases, along with trace gases such as carbon dioxide and ozone, make up the Earth’s atmosphere.^[1]^

Argon is a rare gas, usually considered an inert gas, colorless and odorless, commonly used in industry for purifying steel, filling light bulbs, and welding stainless steel, manganese, aluminum, and other special metals.^[2]^ Normal air contains 0.93%, non-toxic at atmospheric pressure when the concentration of argon in the air reaches 33% or more, can cause asphyxiation.^[3]^ The authors report a case of acute argon poisoning caused by working in an environment containing argon gas.

## 2. Case presentation

A 22-year-old male patient, 175 cm in height and 60 kg in weight, suddenly lost consciousness at 04:50 am 3 days ago while working at the bottom of the furnace chamber in the production plant containing argon gas, and was picked up by a worker and taken away from the bottom of the furnace chamber, then given positive pressure respirator oxygen, at 05:20 am the patient regained consciousness slightly and began to speak, but did not answer questions, called 120 and sent to the local hospital. The patient showed dizziness, headache, weakness, and irritability during the transport, and was referred to the local county hospital. The patient was then transferred to our emergency resuscitation room. The patient was asked about what happened 2 days ago and had impaired memory, sensory retardation, normal cognition, and still had symptoms of dizziness and headache during the period, temperature is 36.6°C, a sinus rhythm of 77 bpm, and blood pressure of 122/69 mm Hg. Respiration is 20 times/minute. There was no specific abnormality in the whole-body examination. There was no special abnormality in the past and family history.

### 2.1. Investigations

On August 20, the local county hospital examination: electrocardiogram (ECG), as well as cranial computed tomography (CT), did not show abnormalities, cranial magnetic resonance imaging (MRI) suggests: bilateral cerebellar hemispheres diffuse slightly high signal, piezoelectric water high signal.

Our emergency resuscitation room examined: complete blood count, arterial blood gas analysis (pH 7.42, PO_2_ 88.7 mm Hg, PCO_2_ 43 mm Hg, HCO_3_^−^ 27.6 mmol/L, SaO_2_ 97.7%), coagulation function, and renal function tests were normal. Potassium ion 3.36 mmol/L (standard range 3.5–5.1 mmol/L) and sodium ion 135.35 (standard range 137–145 mmol/L) suggest electrolyte disturbance with mild hypokalemia and mild hyponatremia. Serum aspartate aminotransferase 81.73 U/L (standard range 17–59 U/L) and serum alanine aminotransferase 134.59 U/L (standard range 21–72 U/L), suggesting hepatic insufficiency.

**Figure 1.**
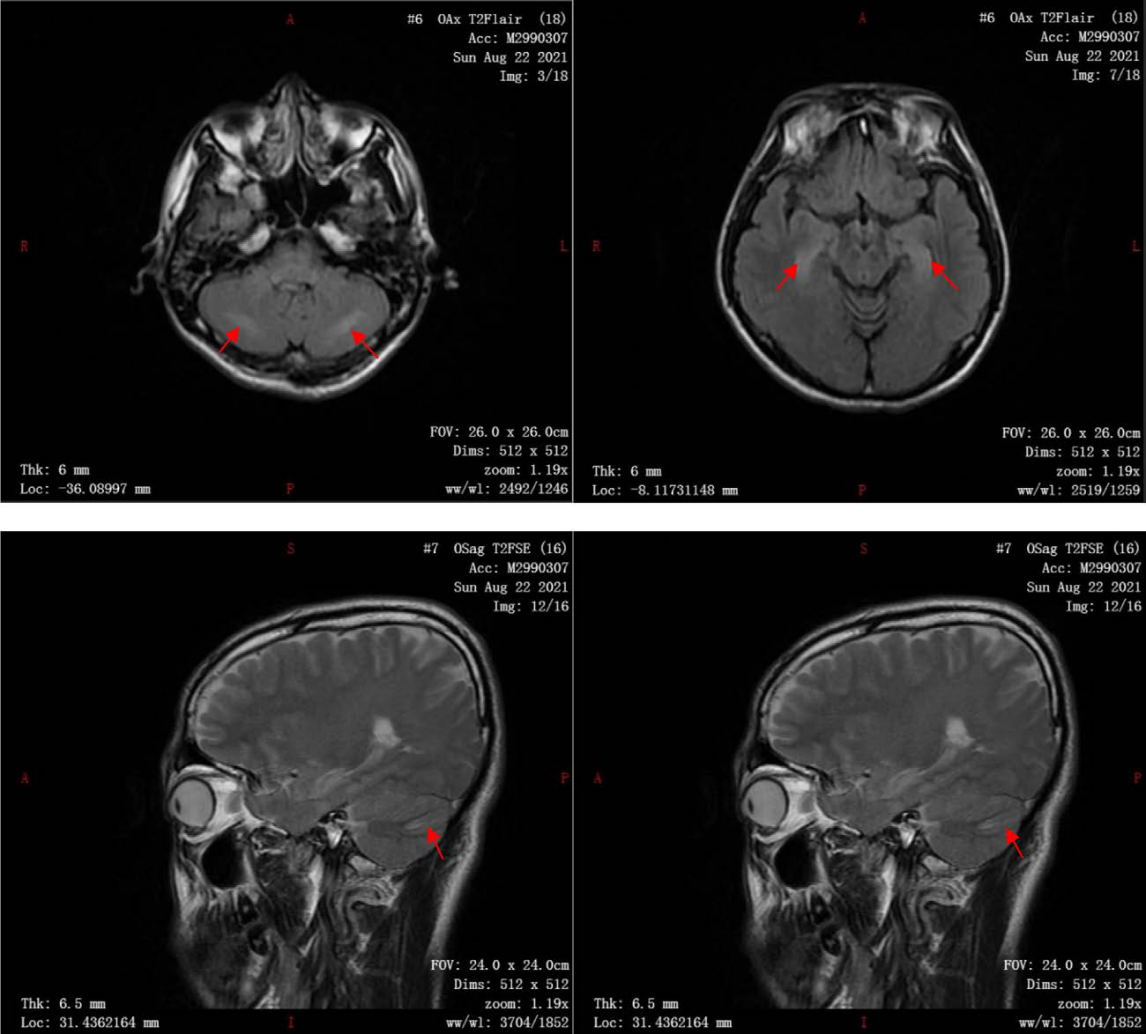
Patches of slightly long T2 signal with asymmetrical distribution in the bilateral cerebellar hemispheres and bilateral hippocampal regions were seen, with slightly high signal in the pressure water and diffusion-weighted imaging (DWI) sequences.

On August 22, after being transferred to the ward, the examination: complete blood count, arterial blood gas analysis (pH 7.39, PO_2_ 149 mm Hg, PCO_2_ 41 mm Hg, HCO_3_^−^ 24.8 mmol/L, SaO_2_ 98.4%), electrolyte examination, liver function examination, and renal function examination all returned to normal. Chest CT examination and cardiac ultrasound examination were normal. Routine 12-lead ECG suggested sinus bradycardia with arrhythmia (Fig. 3), and a 12-lead synchronous dynamic ECG suggested sinus bradycardia (fastest heart rate: 120 bpm, slowest heart rate: 39 bpm, mean heart rate: 53 bpm). Cranial MRI suggested: symmetrical signal abnormalities in bilateral cerebellar hemispheres and bilateral hippocampal regions (Fig. 1). Video electroencephalography (EEG) suggested: that no abnormal waveforms were seen.

**Figure 2.**
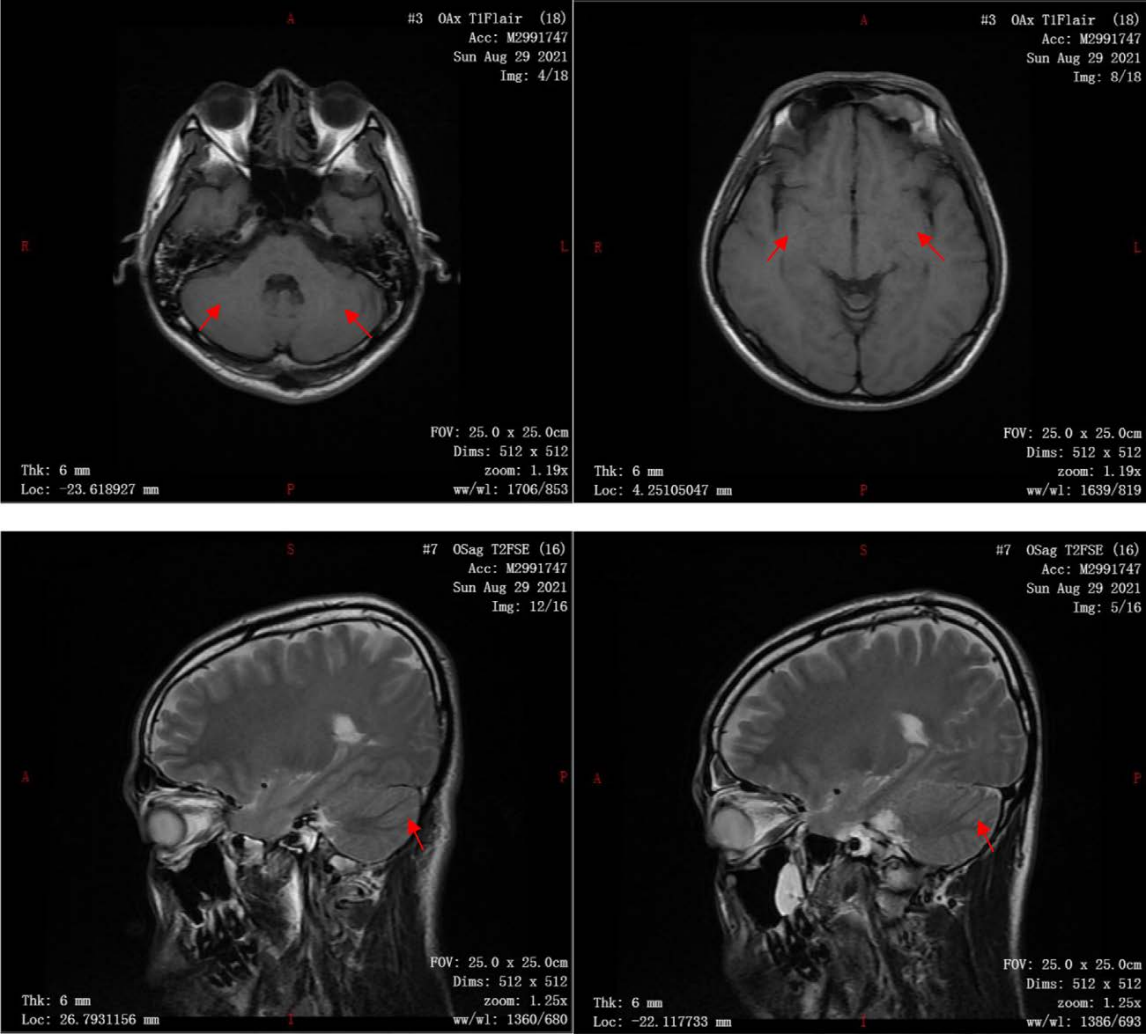
After treatment and re-examination, asymmetrical distribution of patchy slightly longer T2 signal was seen in the hippocampal region bilaterally with better than before, slightly higher signal in the pressurized water and diffusion-weighted imaging (DWI) sequence.

On August 29, the cranial MRI review suggested that the symmetrical abnormal signal in the bilateral hippocampal region was better than before, and the abnormal signal in the bilateral cerebellar hemispheres was not clearly shown this time (Fig. 2). The ECG suggested: sinus rhythm.

**Figure 3.**
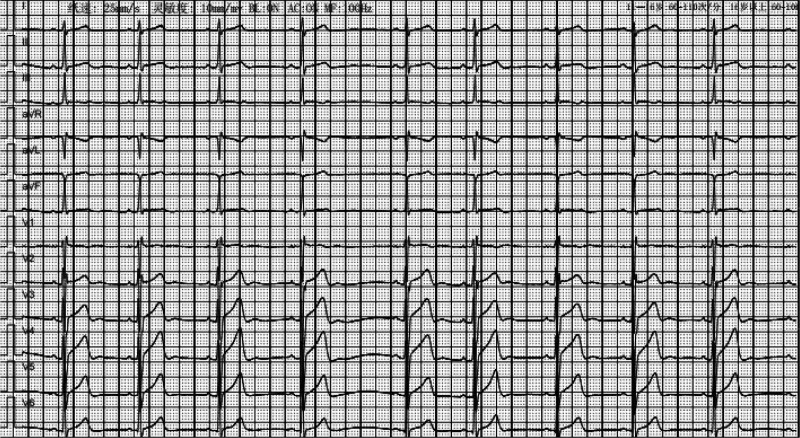
Conventional 12-lead electrocardiogram (ECG): sinus bradycardia with arrhythmias.

### 2.2. Differential diagnosis

There are many causes of loss of consciousness, such as acute cerebrovascular disease, encephalitis, meningitis, diabetic ketoacidosis, hyperosmolar diabetic coma, asphyxiating gas poisoning, and loss of consciousness due to systemic diseases.

• The patient was a young male with no symptoms of hemiparesis, no trauma, no previous history of Transient Ischemic Attack, hypertension, or cardiovascular disease, and no significant abnormalities on cranial CT, which could exclude acute cerebrovascular disease.
• There is no recent history of infection, no fever, no vomiting, no signs of meningeal irritation, no brain parenchymal damage, no abnormal video EEG, and no abnormal CT, so encephalitis and meningitis can be excluded.
• Patients with no history of diabetes or abnormal blood or urine glucose or blood pH and negative urine ketone bodies can be excluded from diabetic ketoacidosis and hyperosmolar diabetic coma.
• Other toxicities, coma caused by systemic diseases were excluded by medical history, relevant clinical manifestations, and auxiliary examinations.

### 2.3. Treatment

Immediately after admission, the patient was treated with nasal cannula oxygen 3 L/min and hyperbaric oxygen therapy once a day. Mecobalamin tablets 500 μg were given orally 3 times a day. Oral Ginkgo biloba extract tablets 40 mg 3 times a day.

### 2.4. Outcome and follow-up

The diagnosis was: asphyxiating gas poisoning (argon gas poisoning), metabolic encephalopathy, and hepatic insufficiency. The patient was discharged on August 31 after treatment with hyperbaric oxygen therapy and nerve-nourishing drugs, with no discomfort, clear consciousness, and good memory, and was followed up by telephone for 2 consecutive months, and the patient is now in good condition with no discomfort.

## 3. Discussion

Argon is a rare inert gas, colorless, odorless, non-toxic in daily atmospheric pressure, nitrogen in the air is 78.09%, oxygen 20.94%, argon contains 0.93%, when the concentration of argon in the air reaches 33% or more, resulting in the reduction of the partial pressure of oxygen in the air, can cause hypoxic asphyxiation of normal people.^[2]^ The degree of asphyxiating gas poisoning is affected by the following factors: the greater the concentration of argon, the longer the exposure time, the heavier the poisoning will be; when accompanied by other toxic gases will enhance the toxicity, such as carbon monoxide, sulfur dioxide, and other toxic gases; in the high temperature and high humidity environment, the existence of anemia, cardiac and cerebral ischemia or insufficient blood supply, fever, and various causes of hypoxemia, will aggravate the condition.

Asphyxiating gas can cause cellular hypoxia, leading to interruption of cellular electron transfer and oxidative respiratory chain, disruption of adenosine triphosphate supply, weakening of adenosine triphosphate-dependent nano-pump activity, the inability of Na^+^ to actively transport outside the cell, leaving the cell in a hyperosmotic state, leading to the entry of water molecules, excessive intracellular water nano-storage, and cellular edema; it can also lead to disruption of intracellular calcium environment, due to impairment of Na^+^ and Ca^2+^ exchange Calcium overload can cause intracellular Ca^2+^ overload, which activates intracellular phosphodiesterase A_2_, leading to the decomposition of cell membrane phospholipids and the production of large amounts of arachidonic acid, which is then converted into thromboxane, leukotrienes, prostaglandins, and other substances, causing inflammatory reactions, microvascular spasm, micro thrombosis, aggravating ischemia and hypoxia, forming a vicious circle.^[4–6]^ Cellular hypoxia also generates a large number of free radicals, which damage the cell membrane structure. As hypoxia leads to the production of oxygen free radicals and induces lipid peroxidation, it can lead to impaired liver function and transient elevation of liver enzymes in patients with mild argon poisoning, and multiple organ insufficiency or failure in severe cases.^[7]^ Asphyxiating gas interferes with the supply, uptake, transport, and utilization of oxygen by cells, making oxygen unable to combine with hemoglobin to form oxygen and hemoglobin for gas exchange in tissues and organs throughout the body, resulting in impairment of the ability of blood to transport oxygen or the ability of tissues to utilize oxygen.^[8]^

In this case report, the patients all experienced loss of consciousness in a higher concentration of argon working environment and woke up with confusion, general malaise, irritability, headache, and memory impairment. Mild argon poisoning manifests as headache, dizziness, nausea and vomiting, and general weakness; moderate manifests as severe headache, nausea, vomiting, and unstable movements and gait. Severe manifestations are hypoxic asphyxia: loss of consciousness, convulsions, coma, incontinence, and even death. Argon, as an inert gas, can cause reduced diffusion of alveolar-capillary membranes, resulting in impaired gas exchange between alveoli and pulmonary capillaries, leading to severe hypoxia.^[9]^ The patient suffers from hypoxic asphyxia in the hypoxic state, and the brain cells are extremely sensitive to ischemia and hypoxia, which causes the accumulation of acidic metabolites and enhances cerebrovascular permeability, resulting in interstitial edema of brain cells, and the accumulation of nasal water in the cells due to the weakened activity of the nano-pump, the disturbance of the intracellular calcium ion environment and the massive production of oxygen radicals caused by brain cell hypoxia, which induces intracellular edema; hypoxia causes the swelling of vascular endothelial cells and The cerebrovascular circulation is impaired by hypoxia, resulting in cerebral infarction, and delayed encephalopathy can occur in a few patients. Argon gas poisoning causes hypoxia in the body and affects the function of brain cells, causing abnormal brain discharge.^[10]^ However, the heartbeat rate was changed and sinus bradycardia appeared, but the patient showed no clinical symptoms, and after treatment, the above symptoms were completely recovered.^[11]^

For argon gas poisoning patients with loss of consciousness, the environmental safety of the rescue site should be fully evaluated before rescue, and rescuers should wear positive pressure air respirators before entering the scene for rescue. Under the condition of ensuring their safety, patients should be quickly removed from the scene of discovery to prevent further aggravation of the disease, placed in an open place with better air circulation, and patients with loss of consciousness should keep their airways open, and if conditions exist for giving If conditions exist for oxygen therapy, 100% oxygen inhalation should be given immediately to accelerate the discharge of argon gas from the body. As in the above case, the patient’s colleague was given oxygen therapy via a respirator. In case of respiratory and cardiac arrest, effective cardiopulmonary resuscitation is performed immediately to improve the hypoxic state and reduce the duration of cerebral hypoxia in a short time. In case of impaired ventilation, a tracheotomy was performed on-site to ensure effective ventilation and oxygenation. Patients were further transported to the hospital for hyperbaric oxygen therapy, cerebral protection therapy (pro-brain metabolism and brain cell energizers, free radical scavengers, neurotrophic drugs, antioxidant Erythropoietin, mitochondrial protector Idebenone), glucocorticoid therapy, symptomatic treatment, and rehabilitation.

### 3.1. Learning points/take home messages

► Argon poisoning causes abnormal changes in the symmetry of the bilateral cerebellar hemispheres and bilateral hippocampal regions of the brain, resulting in altered consciousness, dizziness, and impaired memory.
► As hypoxia leads to the production of oxygen free radicals and induces lipid peroxidation, it can lead to impaired liver function and transient elevation of liver enzymes in patients with mild argon poisoning, and multiple organ insufficiency or failure in severe cases.
► Argon poisoning can cause changes in the heartbeat rhythm rate and can cause sinus bradycardia.
► The EEG often shows severe abnormalities after 1 week, and the EEG performance improves with treatment.
► In terms of treatment early active symptomatic treatment, such as nasal catheter oxygen therapy, high flow and high concentration of oxygen, mechanical ventilation, and hyperbaric oxygen, to correct tissue hypoxia, correct acidosis, scavenge oxygen free radicals, and prevent complications.

## Acknowledgments

LYK was the lead author for this case report, leading the initial patient care and management, and leading the writing of the manuscript. YJZ was the consultant in charge during the case, was actively involved in decision-making and patient treatment, and contributed to the manuscript.

## Author contributions

**Conceptualization**: Yongkai Li.

**Data curation**: Yongkai Li.

**Formal analysis**: Yongkai Li.

**Funding acquisition**: Yongkai Li.

**Investigation**: Yongkai Li.

**Methodology**: Yongkai Li.

**Project administration**: Yongkai Li.

**Resources**: Yongkai Li.

**Software**: Yongkai Li.

**Supervision**: Yongkai Li.

**Validation**: Yongkai Li.

**Visualization**: Yongkai Li.

**Writing – original draft**: Yongkai Li.

**Writing – review & editing:** Yongkai Li, Jianzhong Yang.

## Correction

When originally published, the figures were labelled incorrectly. Figure 1 should have been figure 2. Figure 2 should have been figure 3. Figure 3 should have been figure 1.

## References

[1] Auwarter V Pragst F Strauch H. Analytical investigations in a death case by suffocation in an argon atmosphere. Forensic Sci Int. 2004;143:169–75. 15240039 10.1016/j.forsciint.2004.02.043

[2] Rasanen M. Argon out of thin air. Nat Chem. 2014;6:82. 24345939 10.1038/nchem.1825

[3] Liyun C Chunxia W Shuzhi T. Clinical analysis of 16 cases of acute severe argon gas poisoning. Clinical Focus. 2007;14:1046.

[4] Schito L Rey S. Cell-autonomous metabolic reprogramming in hypoxia. Trends Cell Biol. 2018;28:128–42. 29191366 10.1016/j.tcb.2017.10.006

[5] Semenza GL. Hypoxia-inducible factors in physiology and medicine. Cell. 2012;148:399–408. 22304911 10.1016/j.cell.2012.01.021 PMC3437543

[6] West JB. Physiological effects of chronic hypoxia. N Engl J Med. 2017;376:1965–71. 28514605 10.1056/NEJMra1612008

[7] Nath B Szabo G. Hypoxia and hypoxia inducible factors: diverse roles in liver diseases. Hepatology. 2012;55:622–33. 22120903 10.1002/hep.25497 PMC3417333

[8] Lee P Chandel NS Simon MC. Cellular adaptation to hypoxia through hypoxia inducible factors and beyond. Nat Rev Mol Cell Biol. 2020;21:268–83. 32144406 10.1038/s41580-020-0227-y PMC7222024

[9] Kales SN Christiani DC. Acute chemical emergencies. N Engl J Med. 2004;350:800–8. 14973213 10.1056/NEJMra030370

[10] Mishra OP Delivoria-Papadopoulos M. Cellular mechanisms of hypoxic injury in the developing brain. Brain Res Bull. 1999;48:233–8. 10229330 10.1016/s0361-9230(98)00170-1

[11] Sedmera D Kockova R Vostarek F. Arrhythmias in the developing heart. Acta Physiol (Oxf). 2015;213:303–20. 25363044 10.1111/apha.12418

